# Supplementary material for: Prenylcysteine oxidase 1 like protein is required for neutrophil bactericidal activities
Source: Nat Commun. 2023 May 13;14:2761. doi: 10.1038/s41467-023-38447-z (PMC10182992; doi:10.1038/s41467-023-38447-z)

Supplementary Table 1. List of targeted transcripts

Protein	Protein ID	Function
Serpinb10	Q8K1K6	Unknown
Haus6	Q6NV99	Unknown
Serpinb1a	Q9D154; Z4YK03;Q8VHP7	Serine protease inhibitor
Nedd88	P29595	Protein neddylation
Tmem14c	Q9CQN6	Unknown
Kmt2b	O08550;F6W623;F8WJ40	Epigenetic modifier
Rheb	Q921J2	regulator of mTorc1 pathway
Npm3	Q9CPP0	regulator of chromatin remodeling
Pcyox1l	Q8C7K6;A0A0A6YY26	Unknown
Csfr2b	P26955;P26954	Member of IL-3, IL-5, and GM-CSF signaling

Protein	Fold change GF/SPF PMN	(-)log10(P value)	KO cell lines
Serpinb10	-6.8	4.04	yes
Haus6	-5.9	5.65	yes
Serpinb1a	-3.5	5.43	yes
Nedd88	-4.8	3.64	no
Tmem14c	-3.9	2.16	no
Kmt2b	-3.4	1.71	yes
Rheb	-2.7	1.87	yes
Npm3	-1.6	0.98	yes
Pcyox1l	-3.0	3.76	yes
Csfr2b	-2.2	1.56	yes

Supplementary Table 2. Lentiguide sequences used in the study.

Target Sequence	Barcode Sequence	Gene Symbols	Transcripts
GACACAGGATCAAACGTAAG	GACACAGGATCAAACGTAAG	Haus6	NM_173400.2
AAACTATATGAAAGGCATCA	AAACTATATGAAAGGCATCA	Haus6	NM_173400.2
TTATGTTATGCAAAAATACC	TTATGTTATGCAAAAATACC	Haus6	NM_173400.2
CCCGACGCTGCGTCAAACAG	CCCGACGCTGCGTCAAACAG	Kmt2b	NM_029274.2
GTGAACCCTCTACTCCCCGA	GTGAACCCTCTACTCCCCGA	Kmt2b	NM_029274.2
CCGGGGTGTCTTGATAACAC	CCGGGGTGTCTTGATAACAC	Kmt2b	NM_029274.2
AACAAACTGAATTGTCAATG	AACAAACTGAATTGTCAATG	Rheb	NM_053075.3
ATTCATGACAGAGGACACAA	ATTCATGACAGAGGACACAA	Serpinb1a	NM_025429.2
AATGCTGAAGTGAGCAAACG	AATGCTGAAGTGAGCAAACG	Serpinb1a	NM_025429.2
ATCCCAGAACTGTTGTCTGT	ATCCCAGAACTGTTGTCTGT	Serpinb1a	NM_025429.2
CACCACATTACACTCGTCCG	CACCACATTACACTCGTCCG	Npm3	NM_008723.2
GCACGTGCTGGCGTTGAACA	GCACGTGCTGGCGTTGAACA	Npm3	NM_008723.2
TCAGGAGATTGCAGTTCCTG	TCAGGAGATTGCAGTTCCTG	Npm3	NM_008723.2
TACACTTGGAAGACTGACTG	TACACTTGGAAGACTGACTG	Csf2rb	NM_007780.4
GAAGAAATCGGACAGCTGGG	GAAGAAATCGGACAGCTGGG	Csf2rb	NM_007780.4
TGGAGACTGTAGGCATCCTG	TGGAGACTGTAGGCATCCTG	Csf2rb	NM_007780.4
CAGTGAAAAGCACCACCGAA	CAGTGAAAAGCACCACCGAA	Serpinb10	NM_198028.3
TGAGGTCACGGTTCTGATAG	TGAGGTCACGGTTCTGATAG	Serpinb10	NM_198028.3
TGTCGACTGAGTCATCTGGG	TGTCGACTGAGTCATCTGGG	Serpinb10	NM_198028.3
TGCCGTAGTACCACCAGAGG	TGCCGTAGTACCACCAGAGG	Pcyox1l	NM_172832.4
ACCGCCGGGGAAAATCGGTG	ACCGCCGGGGAAAATCGGTG	Pcyox1l	NM_172832.4
GCAGAACTACGAGAGCGGGG	GCAGAACTACGAGAGCGGGG	Pcyox1l	NM_172832.4
TTTACCAGACGAATTGTACG	TTTACCAGACGAATTGTACG	Cybb	NM_007807.5
CCTCTACCAAAACCATTCGG	CCTCTACCAAAACCATTCGG	Cybb	NM_007807.5
ATTCTAACTTGGATACCTTG	ATTCTAACTTGGATACCTTG	Cybb	NM_007807.5

Supplementary Table 3A. List of prenylated proteins enriched in GCSF-matured WT PMNs compared to *Pcyox1l* CRISPRed PMNs.

Protein names	Gene names	Type of Prenylation	Log(2) Fold Ch	-LOG(P-value)
Cell division control protein 42 homolog	Cdc42	Geranylgeranylation type 1	2.75254753	-1.8215055
Rho-related GTP-binding protein RhoB	Rhob	Geranylgeranylation type 1	3.19885095	-0.5773716
**Ras-related C3 botulinum toxin substrate 1**	**Rac1**	**Geranylgeranylation type 1**	**2.9445417**	-1.8386011
Rho-related GTP-binding protein RhoG	Rhog	Geranylgeranylation type 1	3.05428768	-1.5177444
**Ras-related C3 botulinum toxin substrate 2**	**Rac2**	**Geranylgeranylation type 1**	**1.85856705**	-0.8812755
Ras-related protein Rap-2c;Ras-related protein	Rap2c;Rap2a;Rap2b	Geranylgeranylation type 1	2.03510135	-0.7344001
GTP-binding protein Rheb	Rheb	Geranylgeranylation type 1	1.92246814	-0.5751918
Ras-related protein Rap-1b;Ras-related protein	Rap1b;Rap1a	Geranylgeranylation type 1	2.68866558	-1.5317367
Guanine nucleotide-binding protein G(I)/G(S)/G	Gng12	Geranylgeranylation type 1	2.55431625	-1.6207851
Transforming protein RhoA;Rho-related GTP-bir	Rhoa;Rhoc	Geranylgeranylation type 1	2.32947802	-1.6419738
Ras-related protein Rab-5C	Rab5c	Geranylgeranylation type 2	2.86909383	-1.5738827
Ras-related protein Rab-6A	Rab6a	Geranylgeranylation type 2	2.58116403	-1.7104025
Ras-related protein Rab-21	Rab21	Geranylgeranylation type 2	1.00483721	-0.5470356
Ras-related protein Rab-22A	Rab22a	Geranylgeranylation type 2	1.5646381	-0.7479687
Ras-related protein Rab-24	Rab24	Geranylgeranylation type 2	1.38510805	-0.5991659
Ras-related protein Rab-18	Rab18	Geranylgeranylation type 2	1.35523952	-0.7173535
Ras-related protein Rab-11B;Ras-related protei	Rab11b;Rab11a	Geranylgeranylation type 2	2.65998505	-1.2626944
Ras-related protein Rab-7a	Rab7a	Geranylgeranylation type 2	2.7300966	-1.3273878
Ras-related protein Rab-2A;Ras-related protein	Rab2a;Rab2b	Geranylgeranylation type 2	2.08701157	-1.1250829
Ras-related protein Rab-8A	Rab8a	Geranylgeranylation type 2	2.15197885	-0.9220635
Ras-related protein Rab-5B	Rab5b	Geranylgeranylation type 2	2.73221682	-1.3339274
Ras-related protein Rab-10	Rab10	Geranylgeranylation type 2	2.10590601	-1.4668709
Ras-related protein Rab-8B	Rab8b	Geranylgeranylation type 2	2.24638011	-1.0269791
Ras-related protein Rab-1A	Rab1A	Geranylgeranylation type 2	2.61675725	-1.6751375
Ras-related protein Rab-35	Rab35	Geranylgeranylation type 2	2.38947703	-1.2588383
Ras-related protein Rab-14	Rab14	Geranylgeranylation type 2	2.58291299	-1.0496696
Ras-related protein Rab-31	Rab31	Geranylgeranylation type 2	2.45771104	-1.0418634
Ras-related protein Rab-5A	Rab5a	Geranylgeranylation type 2	3.39175514	-1.3012088
Ras-related protein Rab-1B	Rab1b	Geranylgeranylation type 2	3.54272047	-2.4545457
Ras-related protein Rab-9A	Rab9a	Geranylgeranylation type 2	1.90935513	-0.849204
Lamin-B1	Lmnb1	Farnesylation	2.68364935	-0.9736382
DnaJ homolog subfamily A member 1	Dnaja1	Farnesylation	1.79234602	-1.016483
Ras-related protein Ral-A	Rala	Farnesylation	3.0462695	-1.0650377
Nucleosome assembly protein 1-like 4;Nucleosc	Nap1l4;Nap1l1	Farnesylation	2.95842278	-0.5691443
Guanine nucleotide-binding protein G(I)/G(S)/G	Gng5	Farnesylation	0.92897731	-0.7458928
PC4 and SFRS1-interacting protein	Psip1	Farnesylation	2.67280728	-0.6504927
Synaptobrevin homolog YKT6	Ykt6	Farnesylation	2.8481914	-1.4107345
Ras-related protein Ral-B	Ralb	Farnesylation	4.23374922	-1.3733047

Supplementary Table 3B. List of prenylated proteins enriched in WT progenitors.

Protein names	Gene names	Type of Prenylation	Log(2) Fold Ch	-LOG(P-value)
Cell division control protein 42 homolog	Cdc42	Geranylgeranylation 1	1.28364094	-6.2099741
Ras-related protein Rap-1b;Ras-related protei	Rap1b;Rap1a	Geranylgeranylation 1	2.52261712	-3.0109952
Ras-related C3 botulinum toxin substrate 1	Rac1	Geranylgeranylation 1	3.0891438	-2.4630578
Rho-related GTP-binding protein RhoG	Rhog	Geranylgeranylation 1	2.63960239	-1.3425152
Ras-related C3 botulinum toxin substrate 2	Rac2	Geranylgeranylation 1	1.56550884	-0.9071965
GTP-binding protein Rheb	Rheb	Geranylgeranylation 1	1.69720313	-0.9672456
Guanine nucleotide-binding protein G(I)/G(S),	Gng12	Geranylgeranylation 1	3.3505552	-3.1896463
Transforming protein RhoA;Rho-related GTP-b	Rhoa;4930544G11Rik;Rhoc;Rhob	Geranylgeranylation 1	1.40186843	-1.0526458
Ras-related protein Rab-5C	Rab5c	Geranylgeranylation 2	3.27679204	-2.5973637
Ras-related protein Rab-6A;Ras-related protei	Rab6a;Rab6b	Geranylgeranylation 2	3.05722766	-2.6708138
Ras-related protein Rab-21	Rab21	Geranylgeranylation 2	1.85963083	-0.4779242
Ras-related protein Rab-18	Rab18	Geranylgeranylation 2	2.45197436	-1.2604737
Ras-related protein Rab-11B;Ras-related prote	Rab11b;Rab11a	Geranylgeranylation 2	3.24460884	-2.3067658
Ras-related protein Rab-7a	Rab7a	Geranylgeranylation 2	2.33607252	-1.4297007
Ras-related protein Rab-2A;Ras-related protei	Rab2a;Rab2b	Geranylgeranylation 2	3.19356481	-2.4180093
Ras-related protein Rab-8A	Rab8a	Geranylgeranylation 2	2.62059712	-1.7222823
Ras-related protein Rab-5B	Rab5b	Geranylgeranylation 2	3.18270866	-2.4208945
Ras-related protein Rab-10	Rab10	Geranylgeranylation 2	2.89541107	-2.5077335
Ras-related protein Rab-8B	Rab8b	Geranylgeranylation 2	2.44661162	-1.4248942
Ras-related protein Rab-1A	Rab1A	Geranylgeranylation 2	3.22069376	-2.4929376
Ras-related protein Rab-35	Rab35	Geranylgeranylation 2	2.90012331	-3.4156356
Ras-related protein Rab-14	Rab14	Geranylgeranylation 2	3.18360829	-3.0002612
Ras-related protein Rab-32	Rab32	Geranylgeranylation 2	1.49533315	-1.0304669
Ras-related protein Rab-1B	Rab1b	Geranylgeranylation 2	3.02226613	-2.479686
Ras-related protein Rab-9A	Rab9a	Geranylgeranylation 2	3.02733546	-2.9844337
Lamin-B1	Lmnb1	Farnesylation	1.18412542	-0.9027187
DnaJ homolog subfamily A member 1	Dnaja1	Farnesylation	2.9619837	-1.0671862
Ras-related protein Ral-A	Rala	Farnesylation	2.0768098	-1.0090076
Nucleosome assembly protein 1-like 4	Nap1l4	Farnesylation	2.46986341	-0.3755295
Synaptobrevin homolog YKT6	Ykt6	Farnesylation	2.77775836	-1.9541803
DnaJ homolog subfamily A member 2	Dnaja2	Farnesylation	2.80818152	-2.7287806

Supplementary Figures

Supplementary Figure 1. Heatmap of column correlation by Euclidean distance for proteomic samples show of replicate reproducibly. Neutrophils were purified from non-infected and *P. aeruginosa*-infected SPF and GF SW mice, lysates were trypsin digested, and proteomes solved using LC-MS/MS. Proteome samples were clustered based on Euclidean distance using the 'column correlation' parameter in Perseus. Heatmap range represents reproducibility (as a percentage) across samples with the lowest reproducibility across biological replicates at 84% and highest reproducibility at 94%. The experiment was performed in quadruplicate. SW mice as SPF or GF samples at baseline = uninfected; SW mice as SPF or GF infected = *P. aeruginosa*-infected.

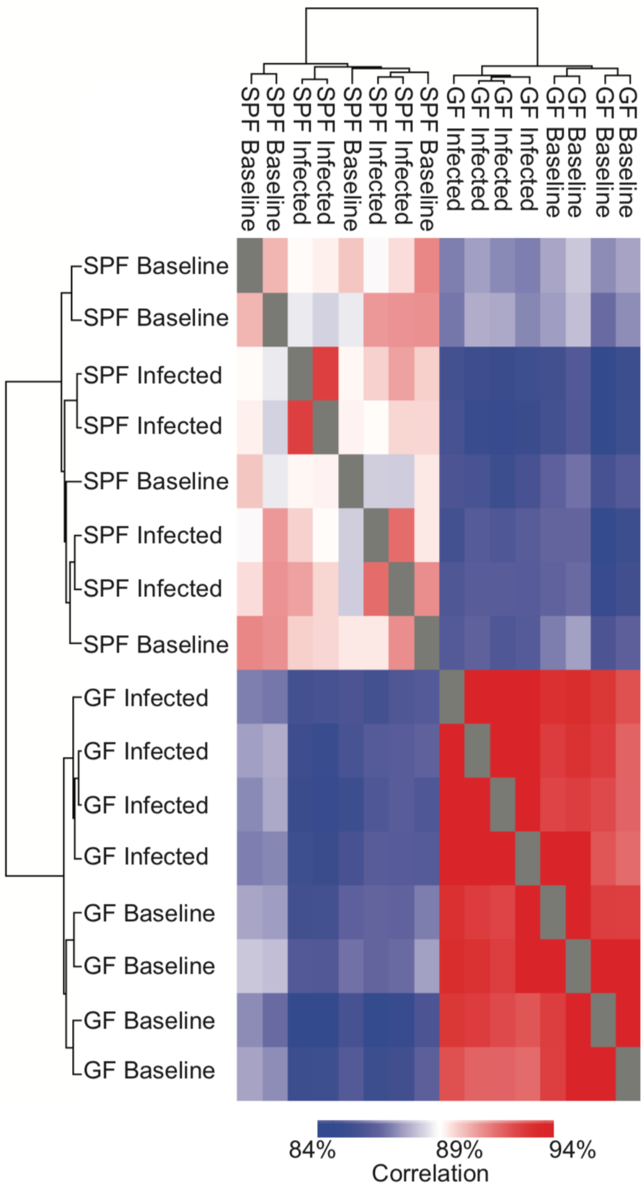
SPF Baseline
SPF Baseline
SPF Infected
SPF Infected
SPF Baseline
SPF Infected
SPF Infected
SPF Baseline
GF Infected
GF Infected
GF Infected
GF Infected
GF Baseline
GF Baseline
GF Baseline
GF Baseline
84%
89%
94%
Correlation

Supplementary Figure 2. Validation of Pcyox1l absence in the generated *Pcyox1l* CRISPRed progenitor cell lines and matured PMNs. a. WB analysis for Pcyox1l presence in WT (control neutrophil cell line, lane 2) and two different *Pcyox1l* CRISPR-ed murine clones (F4 and F6, lanes 3 and 4) where the Pcyox1l expression was CRISPRed out. Protein lysates were harvested from *in vitro* matured with vehicle (FBS) PMNs on day 3. Cell lysates were prepared with RIPA buffer and 8μg total lysate was loaded onto Bis-Tris gel. The membrane was probed with anti-Pcyox1l and hFAB Rhodamine GAPDH primary antibodies. Data are representative of two independent experiments (N=2). **b.** WB analysis for Pcyox1l in WT (control neutrophil cell line) and *Pcyox1l* CRISPR-ed clones (F4 and F6) stimulated with 20 ng/ml GCSF, or 20 ng/ml GCSF with 5 ng/ml GM-CSF. Protein lysates were prepared using matured cells and WB for Pcyox1l was performed. The Pcyox1l and GAPDH WB images are overlayed with the Stainfree image of the same WB to generate composite images. Data are representative of two independent experiments (N=2). Cumulatively, data confirm successful generation of *Pcyox1l* CRISPRed KO PMNs.

a

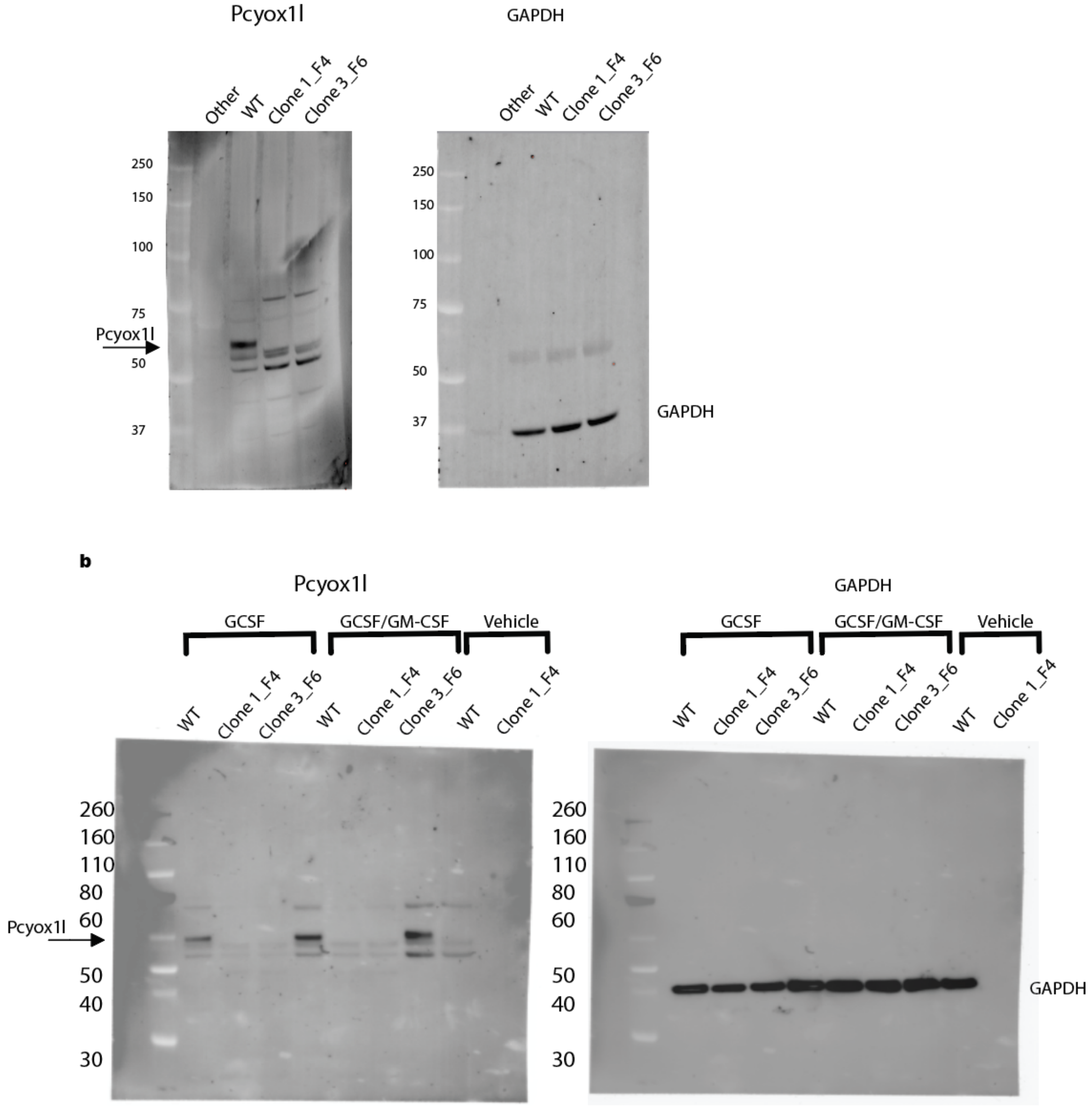
Pcyox1l
GAPDH
Other
WT
Clone 1_F4
Clone 3_F6
250
150
100
75
50
37
Pcyox1l
GAPDH
b
Pcyox1l
GAPDH
GCSF
GCSF/GM-CSF
Vehicle
WT
Clone 1_F4
Clone 3_F6
260
160
110
80
60
50
40
30
Pcyox1l
GAPDH

Supplementary Figure 3. WT and Pcyox1l CRISPRed progenitor cell lines produce multilobed PMNs upon in vitro maturation. WT and *Pcyox1l* CRISPRed PMNs are matured under vehicle (10% FBS), 20 ng/ml GCSF, or 20 ng/ml GCSF with 5 ng/ml GM-CSF for 72 hours. Cell suspensions were spun onto Superfrost slides and stained with Haematoxylin and eosin. Samples were visualized with an Olympus CX23 microscope with a 40X objective. Percentages of cells that displayed multilobed nucleus which is indicative of neutrophils were counted in biological replicas. 1) vehicle-matured WT (open bar, N=21) and *Pcyox1l* CRISPRed PMNs (red bar, N=30), unpaired two-tailed *t*-test with Welch's correction, P<0.0001; 2) GCSF-matured WT (open bar, N=16) and *Pcyox1l* CRISPRed PMNs (red bar, N=25), unpaired two-tailed *t*-test with Welch's correction, P=ns; 3) GCSF and GM-CSF-matured WT (open bar, N=11) and *Pcyox1l* CRISPRed PMNs (red bar, N=24), unpaired two-tailed *t*-test with Welch's correction, P=ns. N=number of biological replica. N.s.=not significant. Data are means +/-SD. Data are presented cumulatively from at least three independent experiments. Source data are provided as a Source Data file. Data verify the predominance of WT banded/multilobed neutrophils in cell cultures upon different maturation conditions.

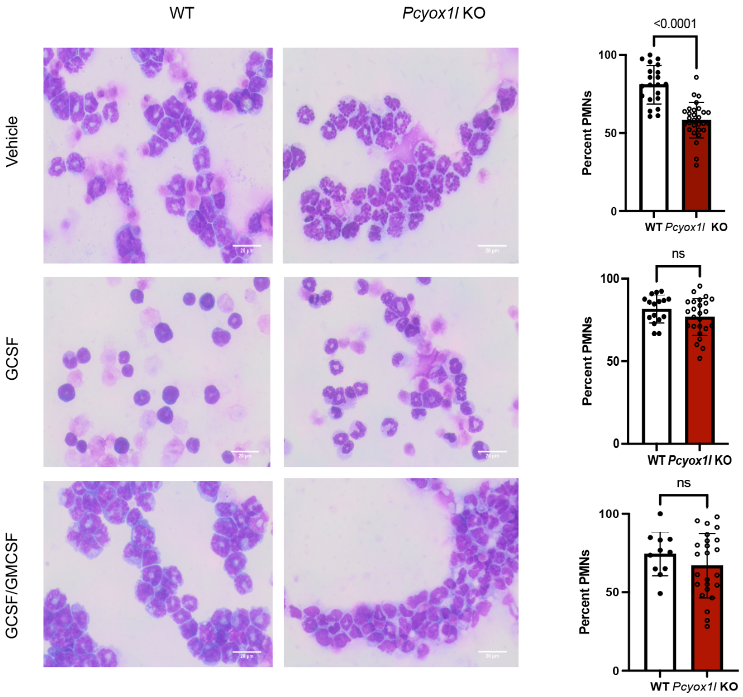
WT
Pcyox1l KO
Vehicle
GCSF
GCSF/GMCSF
<0.0001
ns
ns
Percent PMNs
100
50
0
WT Pcyox1l KO

Supplementary Figure 4. *Pcyox1l* deficiency is associated with reduced PMN bactericidal and opsonophagocytic activities. a. *In vitro*-matured WT (black line, N=4) and *Pcyox1l* CRISPRed PMNs (red line, N=3) was used to measure *Pseudomonas aeruginosa*-induced reactive oxygen species (ROS) release. The cells were dispensed into white 96 well plates (2 × 10^6 cells per well) and incubated with 12.5 uM luminol sodium salt and 5 U of horseradish peroxidase at 37°C for 5 min. After that, the cells were infected with *P. aeruginosa* 6294 at MOI of 10 in the absence of opsonic anti-*P. aeruginosa* MoAb. Luminescence (relative light units, RLU) was measured at 470 nm wavelength. Data are presented as mean values with SD. 2-way ANOVA with P=0.001. Data are representative of three independent experiments (N=3). **b**. *P. aeruginosa*-induced ROS release was measured in *in vitro*-matured WT (black line, N=2) and *Pcyox1l* CRISPRed PMNs (red line, N=2) in the presence of opsonizing antibody. Data are presented as mean values with std. 2-way ANOVA with calculated time and genotype source of variation P=0.001. Data are representative of three independent experiments (N=3). **c.** *Pcyox1l* CRISPRed PMNs show defects in opsonophagocytosis of PAO1. Opsonophagocytic assays with *P. aeruginosa PAO1* was performed in the presence of opsonic anti-*P. aeruginosa* MoAb (anti-Psl). Vehicle, GCSF, and GCSF-GMCSF-matured cells WT (CRISPRed for GFP) (open bars, N=5) and *Pcyox1l* CRISPRed KO (red bars, N=5) clone were incubated with 10% murine serum, anti-*P. aeruginosa* MoAb (28.6 ng/uL), and *P. aeruginosa* PAO1 strain at an MOI of 1:100 for 90 min at 37°C on a rotator. Aliquots taken at 0 and 90 min, serially diluted, and plated on MacConkey agar to determine viable *P. aeruginosa*. Percent killing was calculated as a ratio of colony-forming unit (CFU) at T90 over the spontaneous bacterial growth in the samples without PMNs or T0. Data are mean values and presented with SD. One-way ANOVA, P<0.0001.

Source data are provided as a Source Data file. Cumulatively data show reduced opsonophagocytic killing and ROS release in the absence of Pcyox1l.

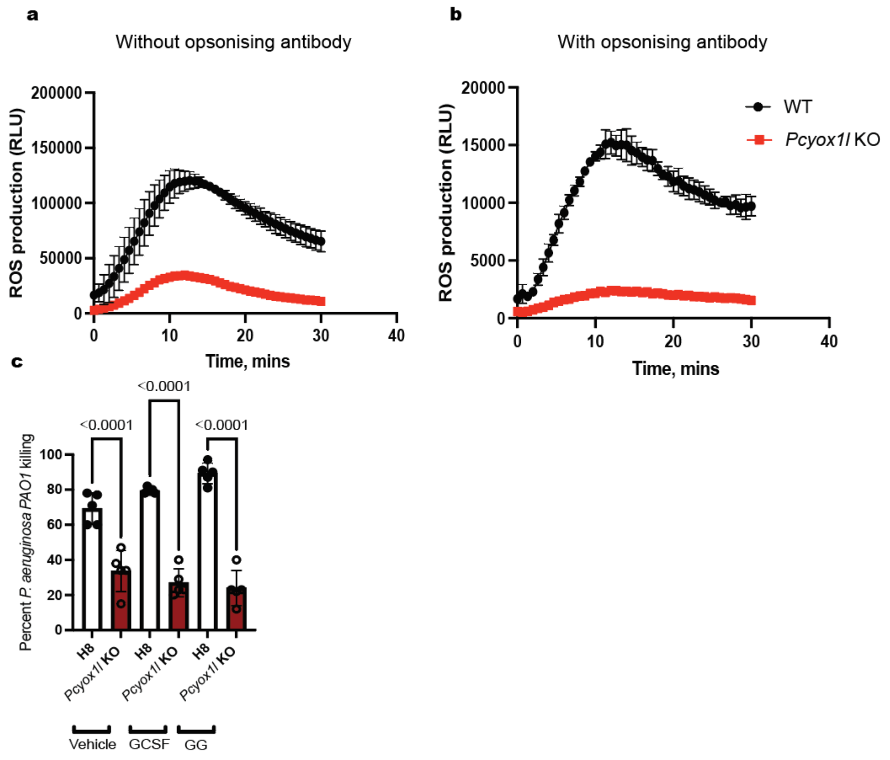

Supplementary Figure 5. ***Pcyox1l*** **KO neutrophils show reductions in prenylated proteins associated with respiratory burst and phagocytosis implicating a connection between PMN functions and prenylation state. a**. Significantly elevated prenylated proteins in WT neutrophils are visualized using publicly available String software and reveal a highly enriched in interactions network (p<1.0e-16). **b**. GO pathway analysis lists the biological processes enriched in the network. The measurement of "strength" describes how large is the enrichment effect. It is calculated as a ratio between the number of proteins in the network versus the number of proteins expected to be associated with this term in a random network. Data show enrichment for proteins involved in respiratory burst (colored in blue, FDR 0.002), phagosome maturation (colored in pink, FDR 0.001), phagosome assembly (colored in yellow, FDR 0.02), and phagocytosis engulfment (colored in green, FDR 0.0002). Cumulatively, the bioinformatic analysis point to defects in phagocytosis consistent with the reductions in *de novo* prenylation of small GTPase at steady state.

a

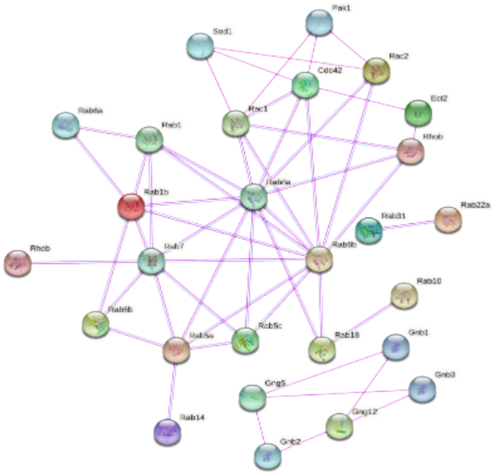

b

GO term	Description	Count in network	Strength	FDR
**GO:0060263**	Regulation of respiratory burst (blue)	2/14	1.94	0.025
**GO:0090382**	Phagosome maturation (pink)	3/22	1.92	0.001
**GO:0001845**	Phagosome assembly (yellow)	2/15	1.91	0.002
**GO:0006911**	Phagocytosis engulfment (green)	4/44	1.75	0.0002

Supplementary Figure 6. BM *Pcyox1l* KO PMNs have detectable Pcyox1 levels. BM-derived PMNs from non-infected and infected with *P. aeruginosa* WT (lanes 1 and 2) and *Pcyox1l* KO (lanes 3 and 4) mice were purified, lysed in RIPA buffer and 25µg total lysate loaded on SDS-PAGE. Samples were loaded twice onto the same gel. The membrane was cut in half and each half was probed with either anti-Pcyox1 (left image) or anti-Pcyox1l (right image) primary antibodies. PageRuler Plus protein ladder served as standard. Images represent uncropped WB data. Data are representative of two independent experiments (N=2). Data show detectable Pcyox1 in all samples including the Pcyox1l KO PMNs.

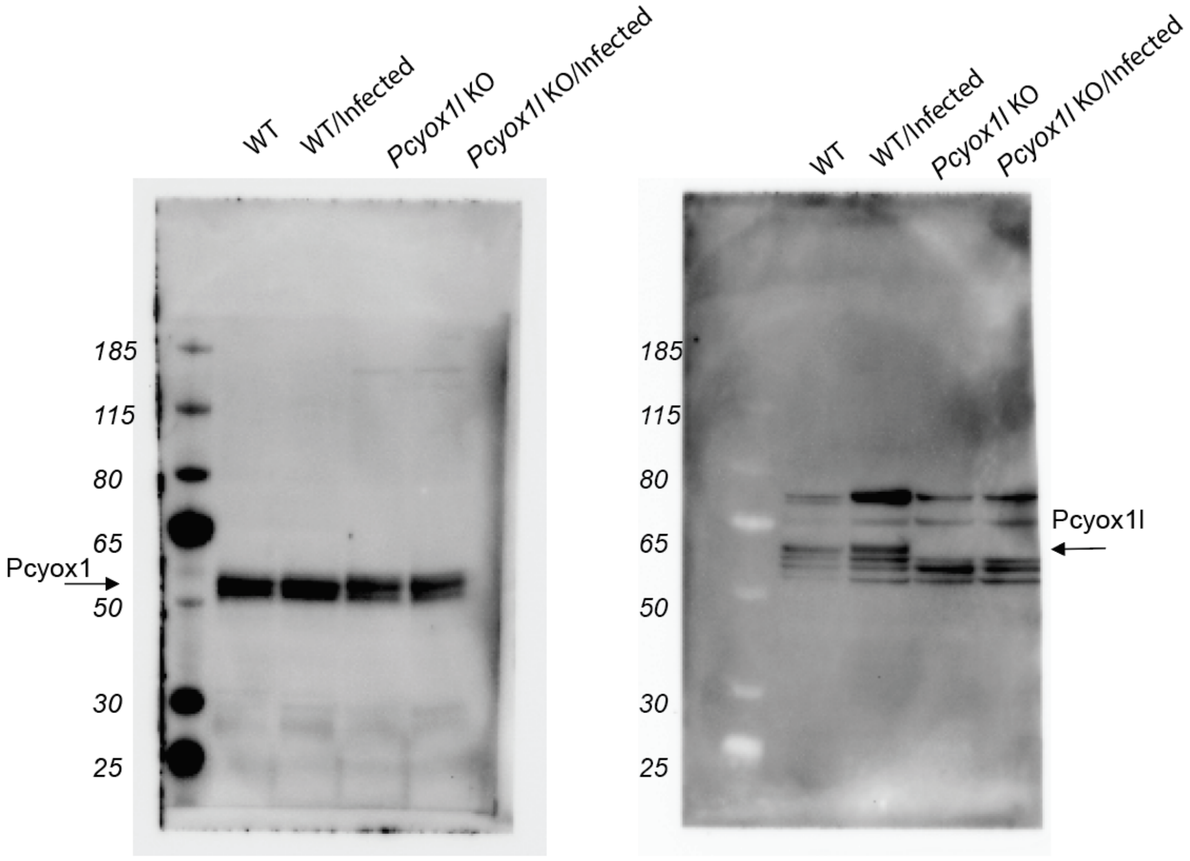

Supplementary Figure 7. Gating strategy for flow cytometry-based analysis of *in vitro* matured WT, *Pcyox1l,* and *Serpin B1a* CRISPRed clones. Viable cells were gated on FSC vs SSC, selecting 7AAD$^-$, Siglec F$^-$, to plot CXCR2 and CD11b. Mature granulocytes were determined as CD11b$^+$, CXCR2$^+$, SiglecF$^-$ cells.

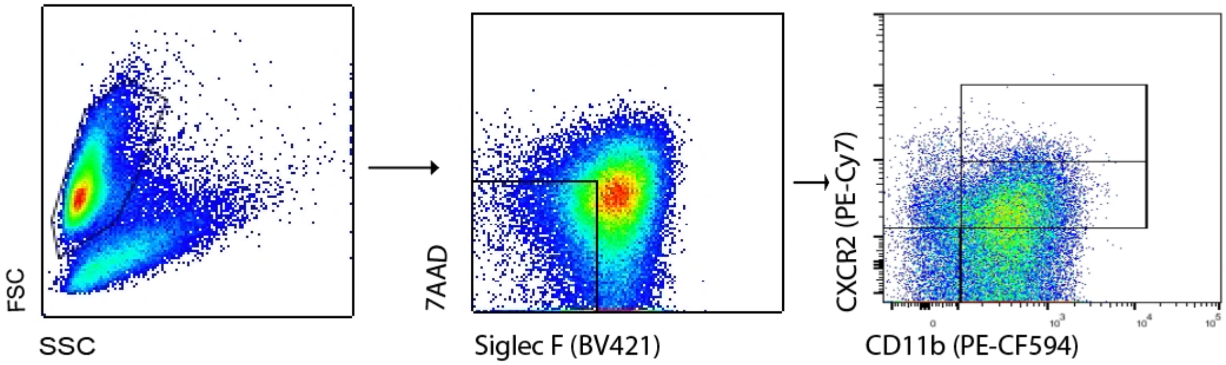

Supplementary Figure 8. ***Pcyox1l*** **CRISPRed neutrophils show diminished viability.**

a. Experimental workflow. WT and *Pcyox1l* CRISPRed GMPs were matured under different conditions *in vitro* including vehicle, 20 ng/ml GCSF and 20 ng/ml GCSF combined with 5 ng/ml GM-CSF. Cells were used for immunophenotyping at either D3 or D5 post-maturation. **b.** WT PMNs were treated with 10 μM cysmethynil for 3 days before viability analysis. Viable cells were quantified using Tryptan Blue exclusion staining. Percent viable cells are plotted. Each symbol represents individual comparisons for cysmethynil-treated vehicle matured WT PMNs (N=4), cysmethynil-treated GCSF matured WT PMNs (N=4), cysmethynil-treated GCSF, GM-CSF matured WT PMNs (N=4). Data are presented as mean values with SD. Ordinary one-way ANOVA with overall P<0.0001 followed by Sidak's multiple comparison test for vehicle- and GCSF-matured PMNs P=0.0001 and P=0.0001 respectively. Data show that inhibition of prenylation by cysmethynil decreases cellular viability of *in vitro* matured WT PMNs, a phenotype similar to that of the *Pcyox1l* CRISPRed PMNs **c.** WT, *Pcyox1l* sand *SerpinB1a* CRISPRed GMPs were matured *in vitro* and immunophenotyped by flow cytometry at D5 post-maturation. Mature neutrophils were defined as viable, SiglecF^{-}, CD11b^{+}, CXCR2^{+}cells. Representative images of histograms from matured WT, *Pcyox1l* KO, and *SerpinB1a* KO PMNs are shown. The bar graph depicts the frequencies of mature PMNs in the cultures. Each symbol presents values from an individual CRISPRed clone, WT (white or grey bars and black symbols, N=2), *Pcyox1l* KO (red bar and symbols, N=3) and *SerpinB1a* KO (white bar and green symbols, N=3) PMNs. Data are presented as mean values with SD. Ordinary one-way ANOVA with Sidak's multiple comparisons test and an overall P=0.0001. Data are representative of two independent experiments (N=2). Source data are provided as a Source Data file. **d**. WB analysis for SerpinB1a in WT and *Pcyox1l* CRISPRed (clone 1 and clone 3). Neutrophil progenitors from

Pcyox1l CRISPRed clones 1 (lanes 1 and 2) and 3 (lanes 3 and 4), Serpin B1a CRISPRRed (lanes 5 and 6) and WT (lanes 7 and 8) clones were matured *in vitro* with 20 ng/ml GCSF (G) and 20 ng/ml GCSF combined with 5 ng/ml GM-CSF (GG) for 3 days. 25µg of total cell lysates were resolved on SDS-PAGE and the WB was probed with anti-SerpinB1a and anti-GAPDH antibodies. The lysates from the *SerpinB1a* CRISPRed KO PMNs which were prepared from cells matured with GCSF (G) or GCSF and GM-CSF (GG) stimulations were used to validate the anti-SerpinB1a antibody immunoreactivity (lanes 5 and 6). The GAPDH WB image is overlayed with the StainFree image of the same gel to generate a composite image. Molecular weight markers in kDa are indicated to the left. Data are representative of two independent experiments (N=2). Uncropped WB images are provided under the WB data section of this document. Data show reduced presence of SerpinB1a in the *Pcyox1l* CRISPRed PMNs. Because SerpinB1a controls PMN viability, it is likely that the relative decrease in SerpinB1a protein levels account for the diminished viability of *Pcyox1l* CRISPRed PMNs. Cumulatively, data show reduced frequencies of matured neutrophils indicative of a significant viability defects in the absence of Pcyox1l, SerpinB1a, and inhibition of prenylation.

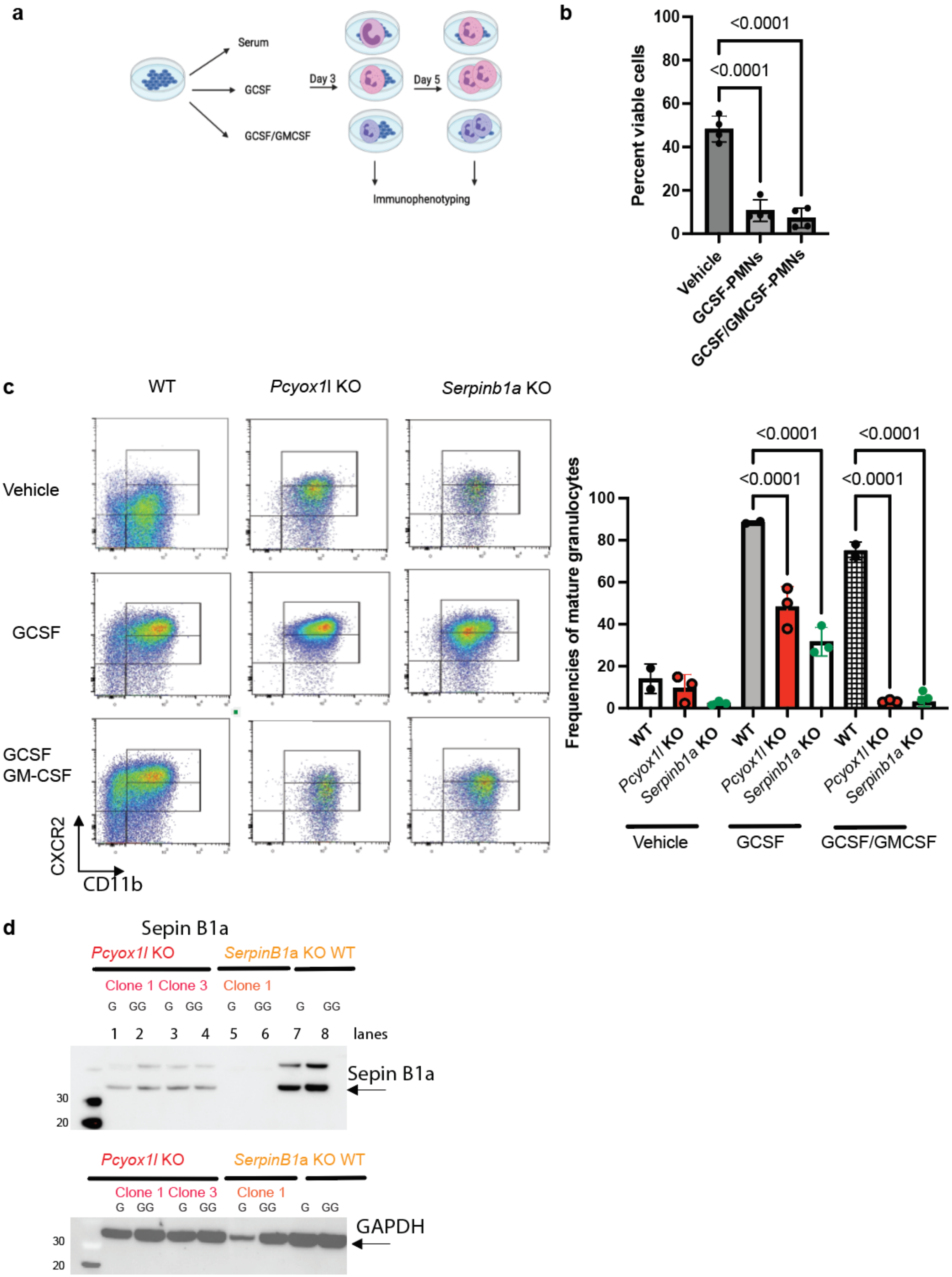

a
Serum
GCSF
GCSF/GMCSF
Day 3
Day 5
Immunophenotyping
b
<0.0001
<0.0001
Percent viable cells
0
20
40
60
80
100
Vehicle
GCSF-PMNs
GCSF/GMCSF-PMNs
c
WT
Pcyox1l KO
Serpinb1a KO
Vehicle
GCSF
GCSF GM-CSF
CXCR2
CD11b
Frequencies of mature granulocytes
<0.0001
<0.0001
<0.0001
<0.0001
0
20
40
60
80
100
WT
Pcyox1l KO
Serpinb1a KO
Vehicle
GCSF
GCSF/GMCSF
d
Sepin B1a
Pcyox1l KO
SerpinB1a KO WT
Clone 1 Clone 3
Clone 1
G GG G GG G GG G GG
1 2 3 4 5 6 7 8 lanes
Sepin B1a
30
20
Pcyox1l KO
SerpinB1a KO WT
Clone 1 Clone 3
Clone 1
G GG G GG G GG G GG
GAPDH
30
20

Supplementary Figure 9. *Pcyox1l* CRISPRed neutrophils show alterations in autophagy. $1x10^6$ neutrophil progenitors were matured under three different conditions: vehicle (10% serum), 20 ng/ml GCSF and a combination of GCSF and 5 ng/ml GM-CSF. Cells were treated with 50 μM autophagy maturation blocker chloroquine (C) or vehicle (DMSO) for 4h on day 3 of maturation. 25μg cellular lysates were examined by WB for LC3 and GAPDH reactivity. Data are representative of two independent experiments with at least two different CRISPR-ed *Pcyox1l* CRISPRed clones to exclude clone-specific effects (N=2). Left panels show WB for LC3 and GAPDH on lysates from WT and *Pcyox1l* CRISPRed PMNs matured in vehicle. The middle panels depict LC3 and GAPDH WB analysis of lysates from WT and *Pcyox1l* CRISPRed matured with 20 ng/ml GCSF (G) stimulations. Right panels depict LC3 and GAPDH WB analysis of lysates from WT and *Pcyox1l* CRISPRed cells matured with GCSF and GM-CSF (GG) stimulations. The presence or absence of chloroquine is indicated as +/-C. The images show non-adjacent lanes on the same WB delineated by black lines. The uncropped WB images are supplied under full size WB images section of this document. Molecular weight markers in kDa are indicated to the left. The GAPDH image is overexposed to show ladder. Ratios of LC3II to GAPDH band intensities were quantified. The quantifications were done using GAPDH densities of non-overexposed bands. Cumulatively, data demonstrate that in the absence of *Pcyox1l*, the autophagy flux is diminished, while the LC3 degradation is elevated in the *Pcyox1l* CRISPRed PMNs.

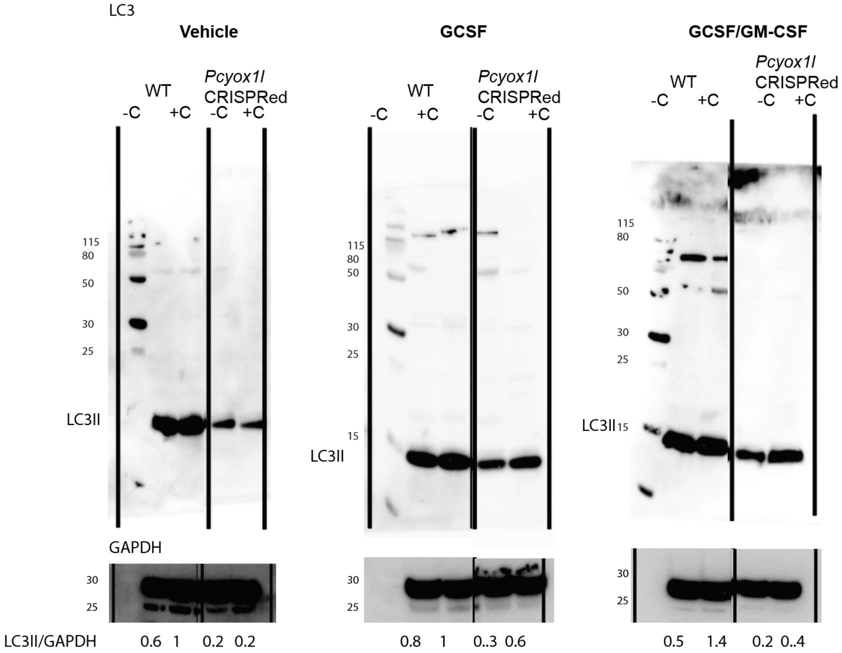

Supplementary Figure 10. *Pcyox1l* CRISPRed neutrophils show decreases in the signaling scaffolding protein p62/SQSTM1, a selective receptor for mitophagy. WB analysis for p62 in lysates from $1x10^6$ WT and *Pcyox1l* CRISPRed-out PMNs matured with vehicle (V), GCSF (G), or GCSF and GM-CSF (GG) stimulations. Protein lysates were harvested from D3 matured cells and 25µg of lysates were loaded on SDS-PAGE. p62 and GAPDH were detected by WB. Molecular weight markers in kDa are indicated to the left. Ratios of p62 to GAPDH band densities were quantified. Data are representative of two independent experiments (N=2). The uncropped WB images are supplied under full size WB images section of this document. Data show significant decreases in p62 levels in the absence of *Pcyox1l*, suggestive of diminished flux to autophagy/mitophagy.

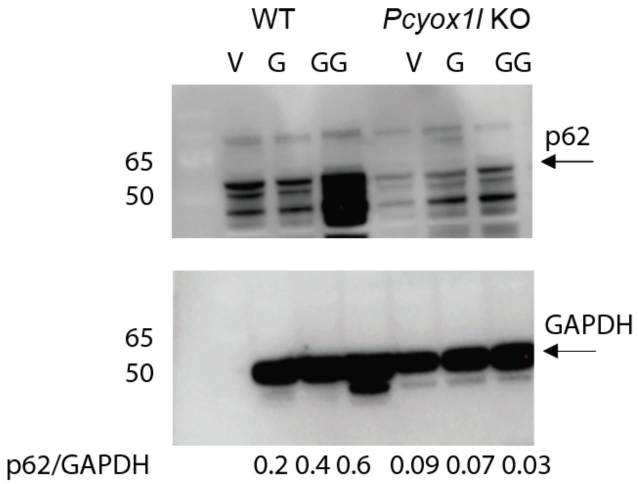
WT
Pcyox1l KO
V G GG V G GG
65
50
p62
65
50
GAPDH
p62/GAPDH
0.2 0.4 0.6 0.09 0.07 0.03

Supplementary Figure 11. Flow cytometry gating strategy for neutrophil characterization in the BM, blood, and spleen. The cell population were gated on singlets, viable cells, $CD45^{+}$, $CD11b^{+}$, $Gr\text{-}1^{+}$ cells.

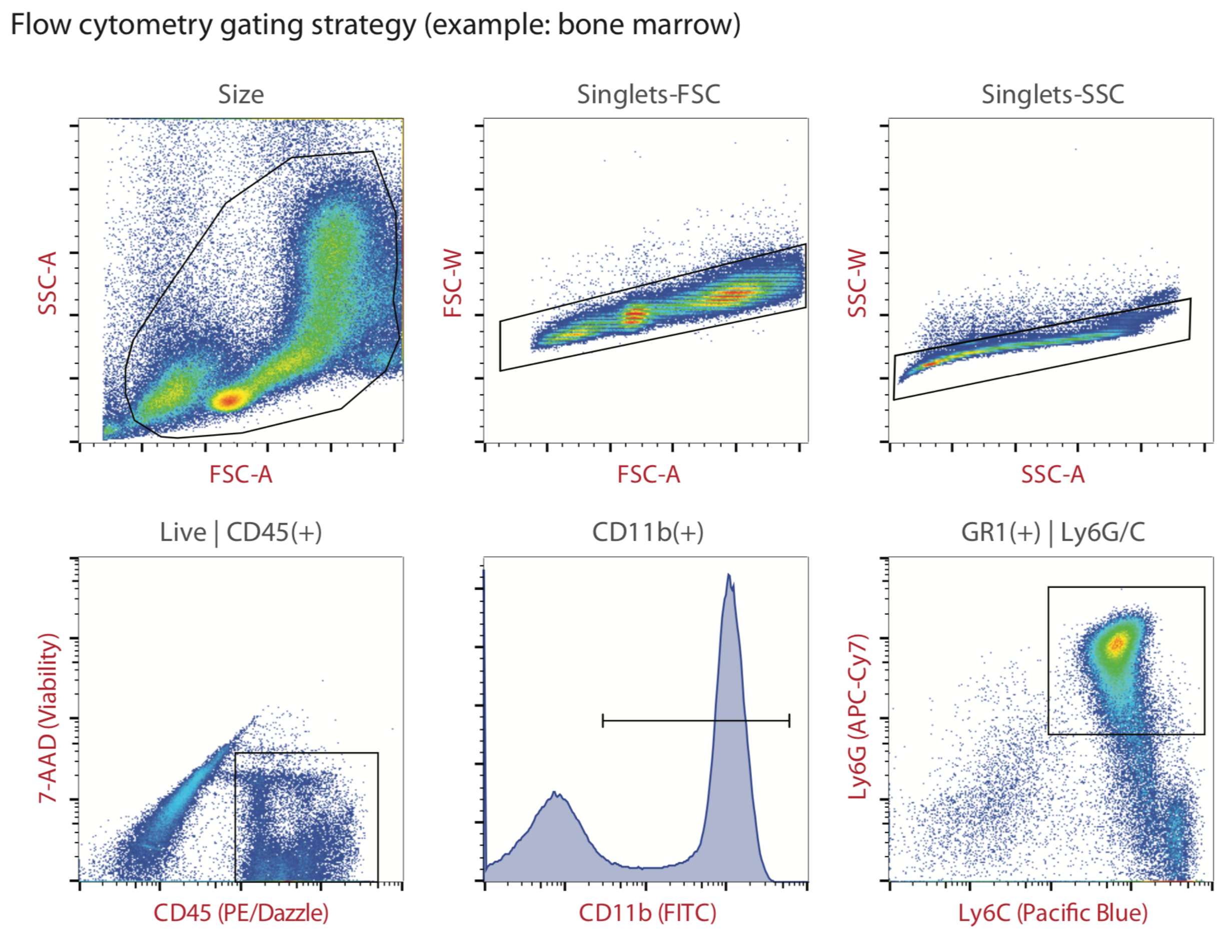

Supplementary Figure 12. Hematopoietic characterization of *Pcyox1l* KO mice under homeostatic conditions

Complete peripheral blood counts of healthy (uninfected) wild-type (blue symbols, N=11) and *Pcyox1l* KO (red symbols, N=11) mice showed no differences in (**a**) white blood cell count, (**b**) hematocrit, or (**c**) absolute neutrophil count. Two-tailed unpaired *t*-test, ns. Flow cytometry was used to enumerate myeloid cells (CD45^{+}, CD11b^{+}) and neutrophils (CD45^{+}, GR1^{+}) in the (**d**) peripheral blood, (**e**) spleen, and (**f**) bone marrow. There was a small but significant reduction in the number of myeloid cells in the bone marrow in the *Pcyox1l* KO mice. Two-tailed unpaired *t*-test, P=0.01. Each symbol presents values from individual mouse. Data are provided as a Source Data file. Data are representative of two independent experiments.

Complete Blood Counts

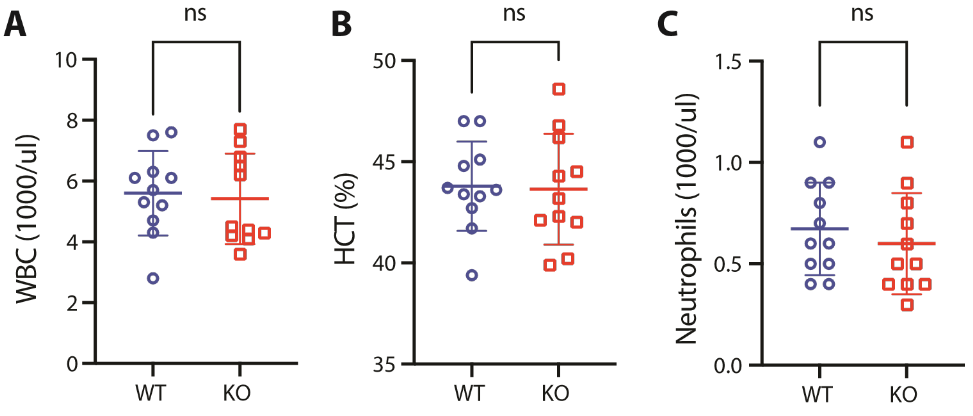

Flow Cytometry

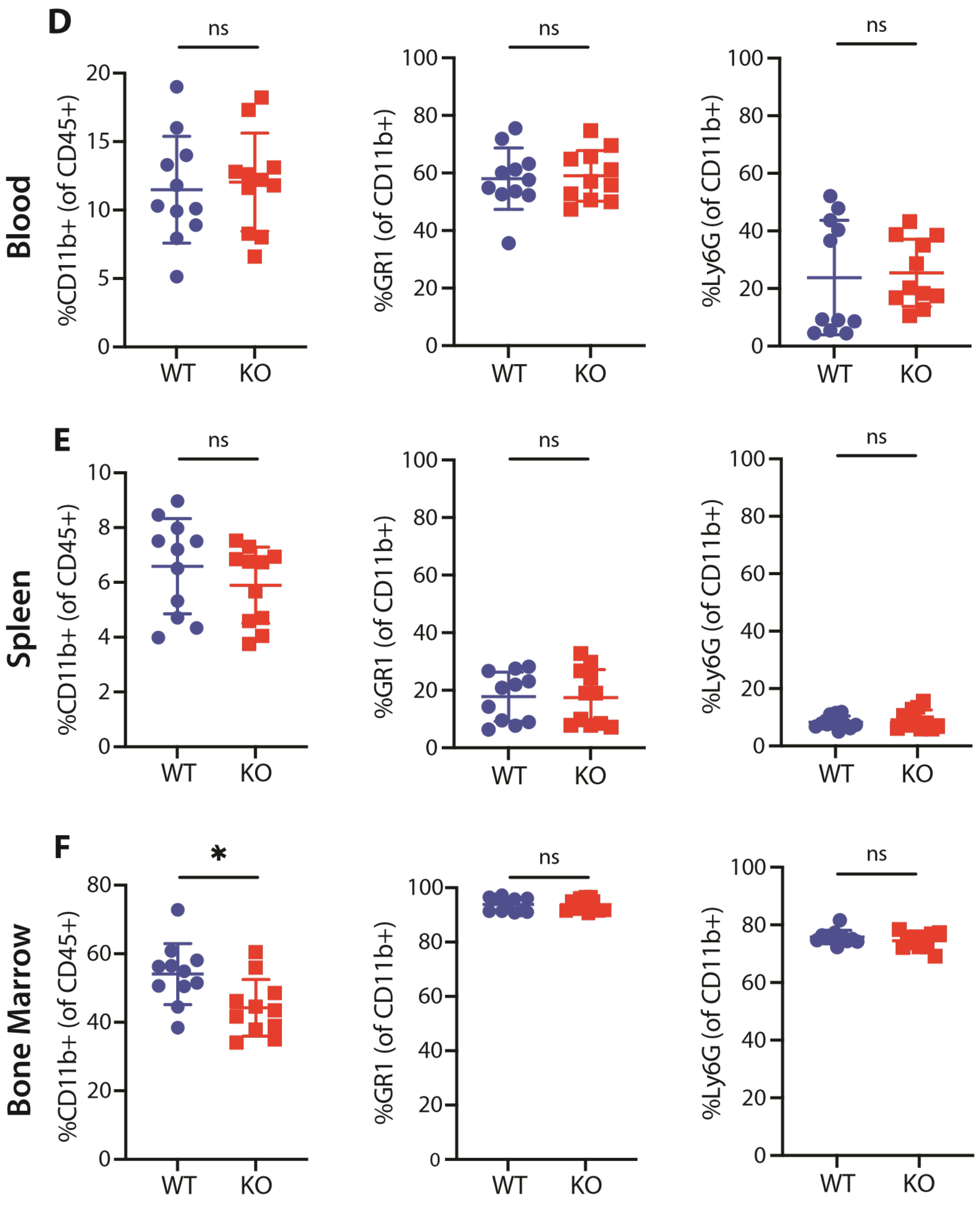
D
E
F
Blood
Spleen
Bone Marrow
%CD11b+ (of CD45+)
%GR1 (of CD11b+)
%Ly6G (of CD11b+)
ns
*
WT
KO

Full size WB images
Suppl. Fig. 7d

Sepin B1a WB

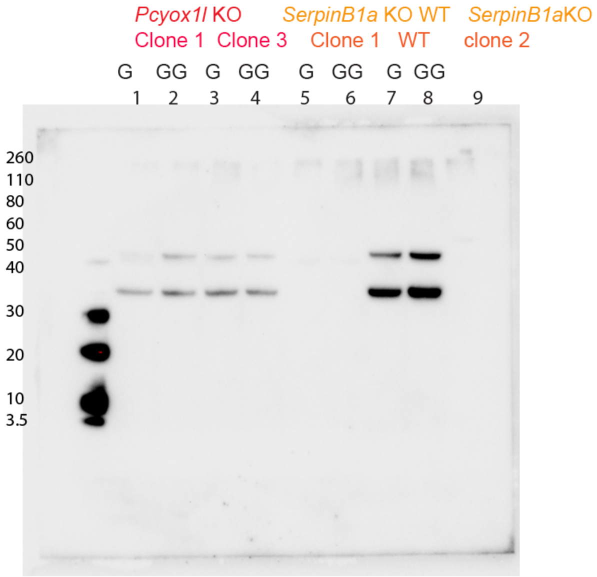

GAPDH WB

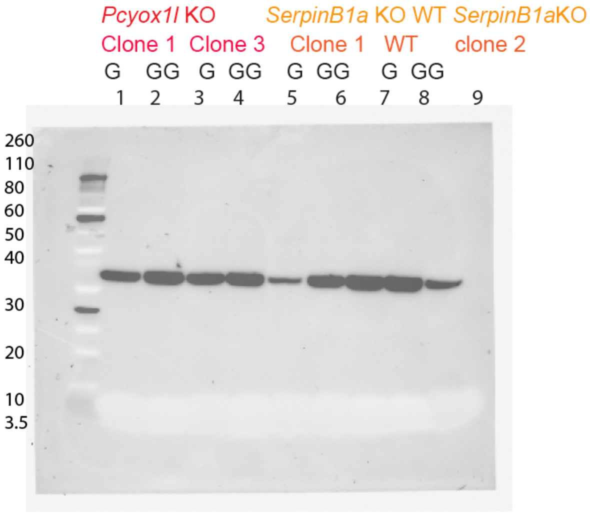

Full size WB Suppl. Fig. 9

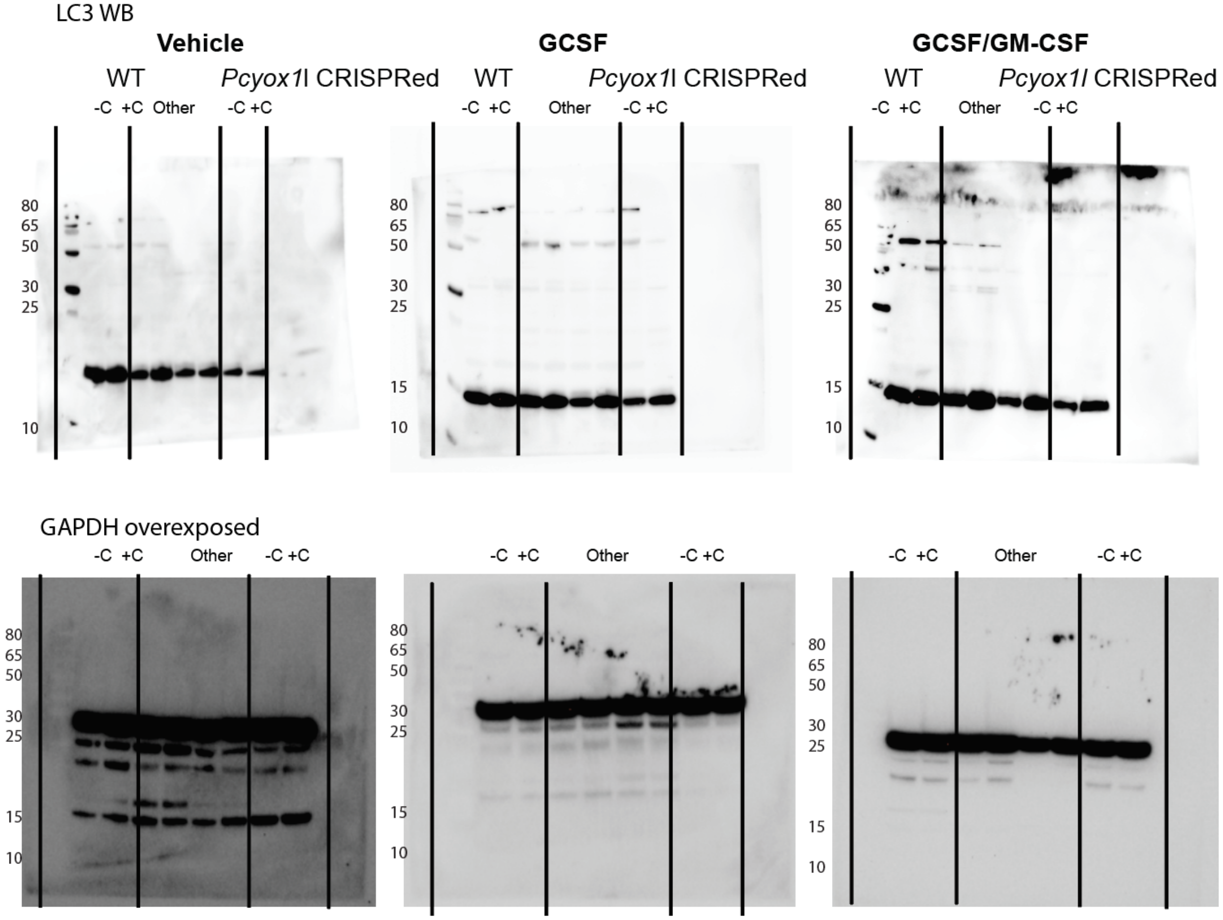

Full size WB Suppl. Fig. 10

WB for p62

WT *Pcyox1l* KO

V G GG V G GG Other

115
80
65
50

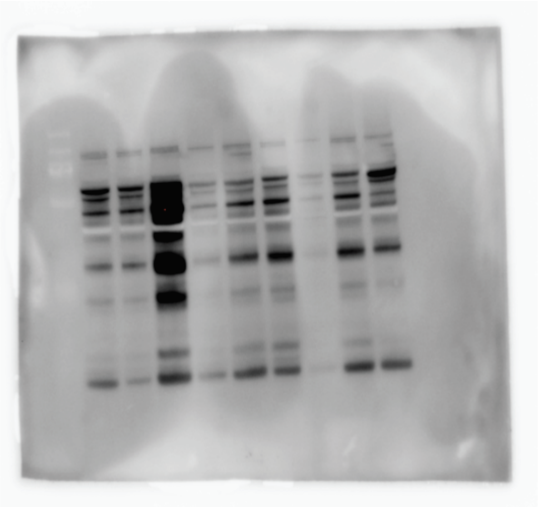

WB for GAPDH

WT *Pcyox1l* KO

V G GG V G GG Other

115
80
65
50

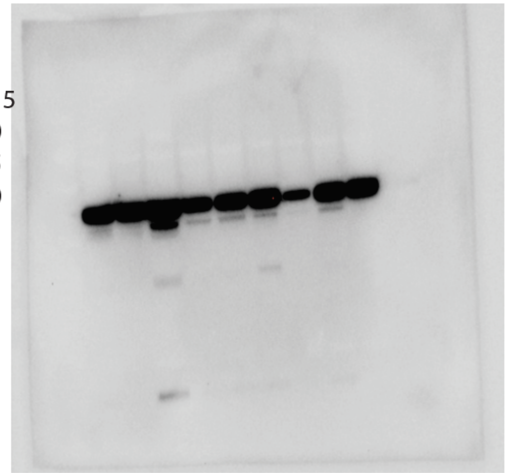

Supplement: Supplementary file 1 — Supplementary Information [file 41467_2023_38447_MOESM1_ESM.pdf]
