# Supplementary material for: Prenylcysteine oxidase 1 like protein is required for neutrophil bactericidal activities
Source: Nat Commun. 2023 May 13;14:2761. doi: 10.1038/s41467-023-38447-z (PMC10182992; doi:10.1038/s41467-023-38447-z)
Supplement: Supplementary file 3 — Reporting Summary [file 41467_2023_38447_MOESM3_ESM.pdf]

nature portfolio

Corresponding author(s): Mihaela Gadjeva

Last updated by author(s): 03/21/2023

Reporting Summary

Nature Portfolio wishes to improve the reproducibility of the work that we publish. This form provides structure for consistency and transparency in reporting. For further information on Nature Portfolio policies, see our Editorial Policies and the Editorial Policy Checklist.

Statistics

For all statistical analyses, confirm that the following items are present in the figure legend, table legend, main text, or Methods section.

n/a	Confirmed	
☐	☒	The exact sample size (*n*) for each experimental group/condition, given as a discrete number and unit of measurement
☐	☒	A statement on whether measurements were taken from distinct samples or whether the same sample was measured repeatedly
☐	☒	The statistical test(s) used AND whether they are one- or two-sided<br>*Only common tests should be described solely by name; describe more complex techniques in the Methods section.*
☐	☒	A description of all covariates tested
☐	☒	A description of any assumptions or corrections, such as tests of normality and adjustment for multiple comparisons
☐	☒	A full description of the statistical parameters including central tendency (e.g. means) or other basic estimates (e.g. regression coefficient) AND variation (e.g. standard deviation) or associated estimates of uncertainty (e.g. confidence intervals)
☐	☒	For null hypothesis testing, the test statistic (e.g. *F*, *t*, *r*) with confidence intervals, effect sizes, degrees of freedom and *P* value noted<br>*Give P values as exact values whenever suitable.*
☒	☐	For Bayesian analysis, information on the choice of priors and Markov chain Monte Carlo settings
☒	☐	For hierarchical and complex designs, identification of the appropriate level for tests and full reporting of outcomes
☒	☐	Estimates of effect sizes (e.g. Cohen's *d*, Pearson's *r*), indicating how they were calculated

Our web collection on statistics for biologists contains articles on many of the points above.

Software and code

Policy information about availability of computer code

Data collection	Flow cytometric data was collected either on a BD Celesta Instrument or Cytek DSP12. LC-MS collections were carried on QExactive HF-X. Prenylomic data was collected with an Orbitrap Fusion Trihybrid mass spectrometer.
Data analysis	Bacterial CFU levels, pathology analysis, metabolite comparisons, and other basic comparisons were done using Prizm v9. Flow cytometry analysis was done using Flowjo V 10.7.1. WB analysis was done using BioRad Imager software. LC-MS analysis was done using MaxQuant (version 1.6.17.0), followed by Perseus.

For manuscripts utilizing custom algorithms or software that are central to the research but not yet described in published literature, software must be made available to editors and reviewers. We strongly encourage code deposition in a community repository (e.g. GitHub). See the Nature Portfolio guidelines for submitting code & software for further information.

Data

Policy information about availability of data

All manuscripts must include a data availability statement. This statement should provide the following information, where applicable:

- Accession codes, unique identifiers, or web links for publicly available datasets
- A description of any restrictions on data availability
- For clinical datasets or third party data, please ensure that the statement adheres to our policy

Data re provided under Source file with the submission. Proteome data sets are uploaded into ProteomeXchange with identifier PXD031115 and PXD009767 (https://www.ebi.ac.uk/pride/archive/projects/PXD030922) and (https://www.ebi.ac.uk/pride/archive/projects/PXD031115).

Human research participants

Policy information about studies involving human research participants and Sex and Gender in Research.

Reporting on sex and gender	N/A
Population characteristics	N/A
Recruitment	N/A
Ethics oversight	N/A

Note that full information on the approval of the study protocol must also be provided in the manuscript.

Field-specific reporting

Please select the one below that is the best fit for your research. If you are not sure, read the appropriate sections before making your selection.

☒ Life sciences ☐ Behavioural & social sciences ☐ Ecological, evolutionary & environmental sciences

For a reference copy of the document with all sections, see nature.com/documents/nr-reporting-summary-flat.pdf

Life sciences study design

All studies must disclose on these points even when the disclosure is negative.

Sample size	Sample size is described under each data set. All cohort predictions were based on Power G analysis to obtain significance level of p<0.05 at 80% probability.
Data exclusions	Data exclusion is described in the Methods section. Animals who displayed wounding at sites different than the infection site were excluded. Animals that displayed infection-nonrelated opacification of either cornea were excluded.
Replication	Infections experiments with the P. aeruginosa clinical isolate 6294 were repeated twice and data are presented cumulatively. Infection experiments with the P. aeruginosa PAO1 strain was done once. Additional replication of the PAO1-induced infection were not carried out as the clinical isolate-induced infections recreated the phenotype of the lab-strain-induced infections.
Randomization	The relative cohort sizes were small negating the use of randomization. Additionally, Pcyox1l KO mice displayed behavioural changes interfering with randomization.
Blinding	CFU and pathology determinations were blinded.

Behavioural & social sciences study design

All studies must disclose on these points even when the disclosure is negative.

Study description	*Briefly describe the study type including whether data are quantitative, qualitative, or mixed-methods (e.g. qualitative cross-sectional, quantitative experimental, mixed-methods case study).*
Research sample	*State the research sample (e.g. Harvard university undergraduates, villagers in rural India) and provide relevant demographic information (e.g. age, sex) and indicate whether the sample is representative. Provide a rationale for the study sample chosen. For studies involving existing datasets, please describe the dataset and source.*

Sampling strategy	*Describe the sampling procedure (e.g. random, snowball, stratified, convenience). Describe the statistical methods that were used to predetermine sample size OR if no sample-size calculation was performed, describe how sample sizes were chosen and provide a rationale for why these sample sizes are sufficient. For qualitative data, please indicate whether data saturation was considered, and what criteria were used to decide that no further sampling was needed.*
Data collection	*Provide details about the data collection procedure, including the instruments or devices used to record the data (e.g. pen and paper, computer, eye tracker, video or audio equipment) whether anyone was present besides the participant(s) and the researcher, and whether the researcher was blind to experimental condition and/or the study hypothesis during data collection.*
Timing	*Indicate the start and stop dates of data collection. If there is a gap between collection periods, state the dates for each sample cohort.*
Data exclusions	*If no data were excluded from the analyses, state so OR if data were excluded, provide the exact number of exclusions and the rationale behind them, indicating whether exclusion criteria were pre-established.*
Non-participation	*State how many participants dropped out/declined participation and the reason(s) given OR provide response rate OR state that no participants dropped out/declined participation.*
Randomization	*If participants were not allocated into experimental groups, state so OR describe how participants were allocated to groups, and if allocation was not random, describe how covariates were controlled.*

Ecological, evolutionary & environmental sciences study design

All studies must disclose on these points even when the disclosure is negative.

Study description	*Briefly describe the study. For quantitative data include treatment factors and interactions, design structure (e.g. factorial, nested, hierarchical), nature and number of experimental units and replicates.*
Research sample	*Describe the research sample (e.g. a group of tagged Passer domesticus, all Stenocereus thurberi within Organ Pipe Cactus National Monument), and provide a rationale for the sample choice. When relevant, describe the organism taxa, source, sex, age range and any manipulations. State what population the sample is meant to represent when applicable. For studies involving existing datasets, describe the data and its source.*
Sampling strategy	*Note the sampling procedure. Describe the statistical methods that were used to predetermine sample size OR if no sample-size calculation was performed, describe how sample sizes were chosen and provide a rationale for why these sample sizes are sufficient.*
Data collection	*Describe the data collection procedure, including who recorded the data and how.*
Timing and spatial scale	*Indicate the start and stop dates of data collection, noting the frequency and periodicity of sampling and providing a rationale for these choices. If there is a gap between collection periods, state the dates for each sample cohort. Specify the spatial scale from which the data are taken*
Data exclusions	*If no data were excluded from the analyses, state so OR if data were excluded, describe the exclusions and the rationale behind them, indicating whether exclusion criteria were pre-established.*
Reproducibility	*Describe the measures taken to verify the reproducibility of experimental findings. For each experiment, note whether any attempts to repeat the experiment failed OR state that all attempts to repeat the experiment were successful.*
Randomization	*Describe how samples/organisms/participants were allocated into groups. If allocation was not random, describe how covariates were controlled. If this is not relevant to your study, explain why.*
Blinding	*Describe the extent of blinding used during data acquisition and analysis. If blinding was not possible, describe why OR explain why blinding was not relevant to your study.*

Did the study involve field work? ☐ Yes ☒ No

Reporting for specific materials, systems and methods

We require information from authors about some types of materials, experimental systems and methods used in many studies. Here, indicate whether each material, system or method listed is relevant to your study. If you are not sure if a list item applies to your research, read the appropriate section before selecting a response.

Materials & experimental systems

n/a	Involved in the study
☐	☒ Antibodies
☐	☒ Eukaryotic cell lines
☒	☐ Palaeontology and archaeology
☐	☒ Animals and other organisms
☒	☐ Clinical data
☒	☐ Dual use research of concern

Methods

n/a	Involved in the study
☒	☐ ChIP-seq
☐	☒ Flow cytometry
☒	☐ MRI-based neuroimaging

Antibodies

Antibodies used	Flow cytometry: 1). Analysis of cell lines: The following antibodies and their corresponding dilutions were used: CD11b PE-CF594 (BD Bioosceinces, cat. 562317, clone M1/70, 1:800), CXCR2 PE-Cy7 (Biolegend, cat. 149315, clone SA044G4, 1:200), SiglecF BV421 (Biolegend, cat. 155509, clone S170071, 1:200).<br>2) Analysis of white blood cells, bone marrow and spleen under homeostatic conditions: The following antibodies and their corresponding dilutions were purchased from BioLegend: CD11b (M1/70, FITC, 1:400), Ly6G (1A8, APC/Cy7, 1:400), B220 (RA3-6B2, Pacific Blue, 1:200), CD3 (145-2C11, APC, 1:100), CD45 (30-F11, PE/Dazzle, 1:400) and Ly6C (HK1.4, BV421, 1:200). 7-AAD was included as a viability dye.<br>3)Analysis of infected corneas: The following markers were used: cKit-FITC (Biolegend, cat. 105805, clone 2B8, dilution 1:200), 7AAD, CD11b-PE (Biolegend, cat. 101207, clone M1/70, 1:200), Ly6C-PE-Cy7 (Biolegend, cat. 128017, clone HK1.4,1:400), CD45-APC (Biolegend, cat. 103111, clone 30F11, 1:400), and Ly6G-BV510 (Biolegend, cat. 127633, clone 1A8, 1:200).<br>4) Western blotting-The proteins were analyzed with the following primary antibodies: polyclonal anti-Pcyox1l (Thermo Fisher Scientific, PA5-25523, Waltham, MA, USA; 1:1000), polyclonal anti–Pcyox1l (Biorbyt, orb35904, Durham, NC, USA; 1:500 dilution), polyclonal anti-SerpinB1a (Aviva Systems Biology, OACD05223, San Diego, CA, USA;1:200), polyclonal anti-LC3 (Sigma-Aldrich, L8918, Saint Louis, MO, USA; 1:200), anti-p62 (Sigma-Aldrich, P0067, 1:200), anti-Glyceraldehyde-3-Phosphate Dehydrogenase clone 6C5 (Millipore, MAB374, Burlington, MA, USA,1:500). Goat anti-rabbit IgG–horseradish peroxidase (Santa Cruz Biotechnology, SC-2004, Dallas, TX, USA; 1:2500) and donkey anti-mouse IgG–horseradish peroxidase (Santa Cruz Biotechnology, SC-2314, Dallas, TX, USA; 1:5000) were used as secondary antibodies. Pcyox1l and GAPDH proteins were also visualized with hFAB Rhodamine GAPDH primary antibody (BioRad, 12004167, Hercules, California, USA; 1:1000) and anti-rabbit StarBrightBlue 700 secondary (BioRad, 12004161, Hercules, California, USA; 1:2500) in the immunofluorescence-based WB analysis .
Validation	polyclonal anti-Pcyox1l (Thermo Fisher Scientific, PA5-25523), polyclonal anti–Pcyox1l (Biorbyt, orb35904), polyclonal anti-SerpinB1a (Aviva Systems Biology, OACD05223) primary antibodies were validated through the use of Pcyox1l and SerpinBa CRISPRed cell line-derived lysates as described in this study. The polyclonal anti-LC3 (Sigma-Aldrich, L8918) was independently validated in PubMedID:24475161 by manufacturer's statement. The anti-p62 (Sigma-Aldrich, P0067 was validated by immunoprecipitation per statement from manufacturer.

Eukaryotic cell lines

Policy information about cell lines and Sex and Gender in Research

Cell line source(s)	The immortalized neutrophil progenitor cell lines are derived from male ER-Hoxb8 C57BL6/N mice.
Authentication	The authentication of the KO cell lines was carried out by WB analysis.
Mycoplasma contamination	Tested negative
Commonly misidentified lines (See ICLAC register)	No commonly misidentified cell lines were used in the study.

Animals and other research organisms

Policy information about studies involving animals; ARRIVE guidelines recommended for reporting animal research, and Sex and Gender in Research

Laboratory animals	The Pcyox1l mouse line 49020 was a product of CRISPR targeting to zygotes, developed by the Knockout Mouse Production and Phenotyping project (KOMP2) at the Jackson Laboratory, and deposited to the MMRRC. Pcyox1l KO mice (line 49020) were cryorecovered and intercrossed to generate Pcyox1l KO and WT littermates for experiments. The Pcyox1l KO are on C57Bl6/J background. 6-9 week-old mice were used for experiments. Additionally, 6-9 week-old mice SPF and GF SW mice were used in the study. The CD18 KO breeders were purchased from Jackson Labs (line 002128). Mice were maintained as breeding pairs at MCP with 12h dark and 12 light cycles with 40-60% humidity and at 65 -75F. Sex as a biological variable was not considered as prior experiments in mice did not point to sex as a determinant in clinical outcomes of P. aeruginosa-induced keratitis. Similarly, there is no sex-bias in human disease severity.
Wild animals	No wild type mice were used in the study.
Reporting on sex	No

Field-collected samples	No field collected samples were used in the study.
Ethics oversight	BWH IACUC protocol 2018N000002

Note that full information on the approval of the study protocol must also be provided in the manuscript.

Flow Cytometry

Plots

Confirm that:

- [x] The axis labels state the marker and fluorochrome used (e.g. CD4-FITC).
- [x] The axis scales are clearly visible. Include numbers along axes only for bottom left plot of group (a 'group' is an analysis of identical markers).
- [x] All plots are contour plots with outliers or pseudocolor plots.
- [x] A numerical value for number of cells or percentage (with statistics) is provided.

Methodology

Sample preparation	1). Cell line analysis: Cells were pelleted and resuspended in 50 µL of FACS buffer (PBS, 5% FBS) containing either a 1:200 dilution of FC block (CD16/32, Biolgened, cat. 101302). To quantify immature and mature neutrophils, the following antibodies were used: CD11b PE-CF594, CXCR2 PE-Cy7, SiglecF BV421, all purchased from Biolegend. 100,000-500,000 events were captured per sample.<br>2). Homeostatic analysis of white blood cells: Peripheral blood was collected via retro-orbital sampling of anesthetized mice. The femurs and spleens were removed following euthanasia. Splenocytes were collected by macerated spleens through a 40-micron filter using the plunger from a 5-ml syringe. Bone marrow mononuclear cells were collected by flushing the femurs and filtering the cells through a 40-micron filter. Hypotonic red blood cell lysis (ACK) was used to deplete red blood cells and the white blood cells were resuspended in FACS buffer (PBS, 2% FBS, 1 mM EDTA) prior to antibody staining for flow cytometry.<br>3) Corneal analysis-1-2x10^6 corneal cells were resuspended in FACS buffer containing Fc block (1:200; Biolegend). To characterize the frequencies of mature neutrophils, the following markers were used: cKit-FITC, 7AAD, CD11b-PE, Ly6C-PE-Cy7, CD45-APC, and Ly6G-BV510 (all from Biolegend). All samples were kept in the dark at 4°C until acquisition where 100,000-500,000 events were acquired for each sample.
Instrument	Cell line phenotypes and corneal infection experiments were carried out on a Cytek DXP12. Analysis of white blood cells under homeostatic conditions was done on a BD Celesta.
Software	The data were analyzed using FlowJo software (version 10).
Cell population abundance	The absolute numbers of infiltrating during infection corneal neutrophils were for WT (N=10, mean 301, stdev=585) and Pcyox1l KO (N=17, mean=4890, stdev=4390).
Gating strategy	See Supplementary figures for gating strategy. Gating strategy for flow cytometry-based analysis of matured WT and Pcyox1l and Serpin B1a CRISPRed clones: Viable cells were gated on FSC vs SSC, selecting 7AAD-, Siglec F-, to plot CXCR2 and CD11b. For corneal flow cytometry experiments, cells were gated on viable, highly granular, CD45APC+, CD11bPE+, Ly6G+, Ly6C+ cells. For bone marrow flow cytometry experiments, cells were gated on singlets, viable, CD45PE/Dazzle+, CD11bFITC+, Gr-1 (Ly6G/C)+.

- [x] Tick this box to confirm that a figure exemplifying the gating strategy is provided in the Supplementary Information.
